# Surgical Operations in Private Practice

**Published:** 1904-11-26

**Authors:** John Aikman


### SURGICAL OPERATIONS IN PRIVATE PRACTICE.

By John Aikman, M.D., C.M., Glas.

### III.—Surgical Interference with the Pleural Cavity.

The discussion opened by Prof. Osler at the last general meeting of the British Medical Association on the treatment of tuberculous pleural effusion and pneumothorax has a very special interest for the general practitioner. Dr. Osler asserted, and after discussion maintained, that early and repeated aspiration gave the best prospect of a successful result. The principle falls into the same line as the suggestions made in my former paper on the treatment of psoas abscess. The most serious contention was the risk of septic instruments and the fear of a mixed infection; this risk should be avoidable. The spread of a tubercular infection from the pleura falls more often under the notice of the general practitioner than the consultant. The former sees the slight pleurisies which give rise at the time to little disturbance of health, and would pass out of recollection if it were not that at a later date there occurs definite infection of some other part of the system; it may be of the opposite lung, or it may be far outside the lung, for the pleura does not seem to exercise the filtration which is the function of the lymphatic glands. It is also more readily paralysed in its resisting power by a mixed infection. The relative frequency of infection from the pleura and lymphatic glands is not a matter for statistics, but I may assert with confidence that the infection from the pleura follows at an earlier date, and is more widely spread. The greater the after care which is given to a slight pleurisy, the stronger will be the tendency to assign to it a rheumatic origin; for we have not yet become accustomed to regard tuberculosis as one of the most frequently cured of all the infections. The more grave the prognosis, the better the result, and no general practitioner can afford to disregard Dr. Osler's signs of danger, "insidious onset, early thickening of the pleura, and early retraction of the affected side." When such signs are present, two things are pretty certain, first that there is little lymph floating loose which would be apt to block an aspirating needle, and secondly, that there is sufficient pressure to cause the infected pleural fluid to exude into the surrounding tissues and infect them. There is no reason for delay, every reason for operation. In acute sthenic cases the operation is better delayed till at least the end of the first week.

Aspiration has always answered well in my experience. The skin should be cleansed by pine oil and ether soap, the needles, and so much of the aspirating apparatus as comes in contact with the fluid, sterilised by boiling, and the hands of the operator cleansed as carefully as the skin of the patient. The point of the needle should be sharp enough to require no preliminary incision of the skin; it should be plunged rapidly through the sixth intercostal space, and retained a minute or more until the blood-vessels wounded by its entrance are sealed by clot. Gentle and slow aspiration is best, it produces less disturbance of the feeble adhesions of the lymph, and minimises the risk of bleeding. It is not easy to say how much of the fluid should be removed, probably two-thirds is enough; but it is certain that it is better to leave a little more fluid than to prejudice recovery by drawing ever so little blood by allowing the end of the needle to come into contact with the visceral pleura. It is well to wipe the portion of the canula outside the skin with an antiseptic cloth before removing it; the skin may then be pinched up around it by the thumb and finger of the left hand and kept compressed for a minute or so after it is withdrawn. A pad of double cyanide gauze, fixed by plaster, is the best dressing; and it is good practice to fix the arm to the side for at least twenty-four hours. A very few days suffice to restore quiet within the pleural cavity, and in view of a possible tubercular origin, a return to an outdoor life is advisable at an early date.

There is, however, a more pressing case in which the general practitioner may be called upon to take active measures. When there is an antecedent measles, whooping cough, or diphtheria, he is on his guard against the occurrence of a lobular pneumonia; but there is a fever of childhood attended by rapid breathing, a running pulse, a foul tongue and a fœtid breath, with extraordinary fœtid stools, which he is apt to explain by disorder of the primæ viæ, and rightly, but which is attended by the formation of toxins which attack the lung in the form of lobular pneumonia and sometimes lead to the sudden filling of the pleural cavity. There are always local signs of pleurisy, small in extent, sometimes barely beyond the size of the end of the stethoscope; bronchial breathing, high pitched and near the ear, a slight friction sound mixed with crepitation, and the whole very sharply defined. There is often tenderness to pressure over a limited area. If the disease has been recognised, he is not startled at the sudden occurrence of a great extension of dulness, with urgent signs of dyspnœa. It is well to note that the dulness is the most important sign, for the puerile breathing in the state of struggle comes loudly through the layer of fluid and is misleading. There is often a fall of temperature and signs of collapse. That is not the time for operative interference; digitalis, strychnia and musk rally the little patient and next day the fever is much less and the state relieved. A grain or two of calomel is a good antiseptic and gentle aperient, and rest limits the extension of the fluid. But the case must be watched; I have seen recovery result without resort to surgical means, and, on examination some years later, have found the residual damage greater than after treatment by incision and drainage. I have usually been able to locate the site of this disease in front of the posterior axillary line, and most often much in front of it; it would almost seem as if it bore some relation to the distance of the part from the blood supply at the root of the lung. About two days after the acute symptoms the patient is ready for incision; the skin must be carefully purified, and the hands of the operator, and the knife used to make a clean cut down to the costal pleura, near the upper margin of the rib which is the lower limit of the dulness. This site has the advantage of provoking less bleeding, and leaving a greater padding of muscle between the drainage tube and the intercostal nerve. The pleura may now be examined and, if needful, pricked; only to be followed by free incision. No antiseptic should be used in the deeper parts of the wound: I have never seen embolism follow a gentle washing with boiled water, though I refrain in acute cases from that, but I have seen smoky urine follow the use of a 5 per cent. solution of phenol in the deeper parts of the wound. Good drainage is secured by an indiarubber tracheotomy tube with a window cut out of its greater curvature. The tube should be boiled before use. Two tubes should be in use for service alternately, and the used tube should be cleansed with ether soap and boiled before being used again. After three days there is no objection to gentle flushing, loose lymph comes away, and by the end of the second week the drainage tube may be removed. Double cyanide gauze covered by salicylic wool is a good dressing; it should be changed frequently. When the drainage tube is removed, I use salicylic wool next to the wound for two or three days. I have noted that it has a destructive effect on the most recently formed tissue and keeps the sinus open, but this heals with wonderful rapidity when a milder dressing is substituted. These remarks apply, with an allowance for scale, to the larger empyemata.

Pyopneumothorax is a very grave disease which can sometimes be brought within reach of better treatment by simple surgical measures. Aspiration is not good practice, it is apt to suck the lung against the canula; but with strict aseptic precautions the following is useful:—Cleanse a trocar and canula by boiling, also a piece of Batiste cloth half-an-inch broad by one and a half inches long; thrust the trocar through the lower half of the cloth and puncture. As you withdraw the trocar let the flap of cloth fall over the opening of the canula. It is not difficult to insure that this acts as a valve until the air is exhausted, and after a little quiet, to pack it in an aseptic dressing so that the effect will be continuous. Under such treatment the aperture in the lung may heal and the case resolve itself into the simpler form of an empyema.

